# Changes in the Eye Microbiota Associated with Contact Lens Wearing

**DOI:** 10.1128/mBio.00198-16

**Published:** 2016-03-22

**Authors:** Hakdong Shin, Kenneth Price, Luong Albert, Jack Dodick, Lisa Park, Maria Gloria Dominguez-Bello

**Affiliations:** a Division of Translational Medicine, New York University School of Medicine, New York, New York, USA; b Department of Ophthalmology, New York University School of Medicine, New York, New York, USA

## Abstract

Wearing contact lenses has been identified as a risk factor for the development of eye conditions such as giant papillary conjunctivitis and keratitis. We hypothesized that wearing contact lenses is associated with changes in the ocular microbiota. We compared the bacterial communities of the conjunctiva and skin under the eye from 58 subjects and analyzed samples from 20 subjects (9 lens wearers and 11 non-lens wearers) taken at 3 time points using a 16S rRNA gene-based sequencing technique (V4 region; Illumina MiSeq). We found that using anesthetic eye drops before sampling decreases the detected ocular microbiota diversity. Compared to those from non-lens wearers, dry conjunctival swabs from lens wearers had more variable and skin-like bacterial community structures (UniFrac; *P* value = <0.001), with higher abundances of *Methylobacterium*, *Lactobacillus*, *Acinetobacter*, and *Pseudomonas* and lower abundances of *Haemophilus*, *Streptococcus*, *Staphylococcus*, and *Corynebacterium* (linear discriminant analysis [LDA] score = >3.0). The results indicate that wearing contact lenses alters the microbial structure of the ocular conjunctiva, making it more similar to that of the skin microbiota. Further research is needed to determine whether the microbiome structure provides less protection from ocular infections.

## IMPORTANCE

As in other body sites (i.e., the gut, skin, and mouth), the eye has a normal community of bacteria which are expected to confer resistance that provides protection from invaders. However, the eye microbiome has been largely neglected and is relevant to eye health and understanding eye diseases and to discovery of its functions. This report of a baseline study shows differences in the eye microbiome of contact lens wearers in relation to those of non-lens wearers and has the potential to help future studies explore novel insights into a possible role of the microbiome in the increased risk for eye infections in contact lens wearers.

## INTRODUCTION

In culture-dependent studies, over 50% of swabs from the conjunctiva showed growth of skin-like bacteria (mostly coagulase-negative staphylococci, *Propionibacterium*, and *Corynebacterium*) (1). Ocular bacterial communities have been studied using culture-dependent methods (2, 3) and, more recently, with 16S rRNA gene sequencing in healthy subjects (4) and in people with eye diseases (5, 6). Using sequencing methods, additional bacteria, such as *Pseudomonas*, *Bradyrhizobium*, *Acinetobacter*, *Brevundimonas*, *Aquabacterium*, *Sphingomonas*, *Streptococcus*, *Streptophyta*, *Methylobacterium*, *Enhydrobacter*, *Bacillus*, and *Ralstonia* spp., were detected (4–6). As in other body sites, the ocular microbiota is expected to play a defensive role against colonization of pathogens in the eye (7). Despite being important in ophthalmology, the eye microbiome has been largely neglected, and its functions remain unknown.

In the United States, over 30 million people wear contact lenses, nearly one-third of the ~100 million worldwide (8). Wearing contact lenses has been identified as a risk factor for eye conditions such as giant papillary conjunctivitis (9) and keratitis (10–12). The conjunctiva of patients with vernal keratoconjunctivitis (inflammation of the eye that involves both the cornea and conjunctiva) showed increased Toll-like receptor 4 (TLR-4) levels in relation to those of controls (7), supporting the hypothesis of microbial involvement and interaction with host conjunctival epithelium.

While several studies have used culture-dependent approaches to demonstrate bacterial contamination of contact lenses (13, 14), little is known about the impact of contact lenses on the structure and function of the microbiota on the ocular surface. In this work, we compare the microbiota of the ocular surface of lens wearers with that of non-lens wearers using 16S rRNA gene sequencing surveys.

## RESULTS

We obtained 7,010,096 sequences (paired end; Phred ≥ Q20) with an average of 21,569 reads per sample, yielding 11,750 operating taxonomic units (OTUs) (11,700 OTUs without singletons) (see Table S1 in the supplemental material).

The bacterial alpha diversity of the conjunctiva was significantly higher in subjects sampled at the laboratory without anesthetic than was seen in the ophthalmologic practice, which used an anesthetic eye drop (whole-tree phylogenetic diversity [PD], number of observed species; *P* value = <0.001 [nonparametric Student’s *t* test]) (see Fig. S1A in the supplemental material). Using an anesthetic eye drop significantly altered microbial community composition (linear discriminant analysis [LDA] score = >3.0; see Fig. S1B) and structure (permutational analysis of variance [PERMANOVA] *P* value = <0.001; see Fig. S1C), as measured by unweighted/weighted UniFrac distances. In addition, the use of contact lenses caused only minor changes in the conjunctival microbiota of subjects at the ophthalmology practice (see Fig. S2), supporting the idea of an effect of anesthetic. Due to the apparent perturbation caused by the eye drops, we performed further analyses solely on the 20 subjects sampled in the laboratory without anesthesia.

**FIG 1:**
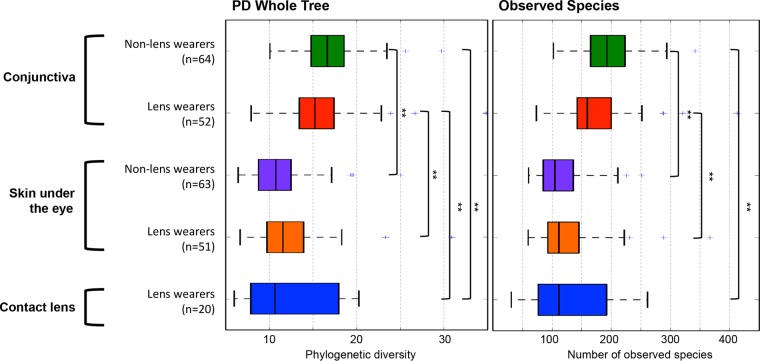
Bacterial diversity in the conjunctiva, skin, and contact lens between non-lens wearers and lens wearers. Box plots of alpha diversity were generated with rarefication to 2,090 reads per sample. The nonparametric *P* values were calculated using 999 Monte Carlo permutations. +, outlier samples excluded from the analyses; **, *P* value = <0.05.

A total of 250 samples obtained in the laboratory (116 conjunctiva, 114 skin under the eye, and 20 contact lenses) were rarefied at 2,090 sequences per sample (see Table S2 in the supplemental material). Notably, the conjunctival samples showed higher alpha diversity than the skin under the eye or contact lenses (*P* value = <0.05 [nonparametric Student’s *t* test]; Fig. 1). In the same sequencing run in which we sequenced the eye project samples, we sequenced samples of the vaginal microbiota from different subjects. The alpha diversity of the vaginal microbiota was similar to that reported by the HMP Consortium (15), and the bacterial diversity in the skin under the eye in our study was similar to the bacterial diversity in the skin of the face reported by Bouslimani et al. (16) (see Fig. S3A). We consider these good positive controls and are confident in the diversity found in the conjunctiva and skin under the eye.

**FIG 2:**
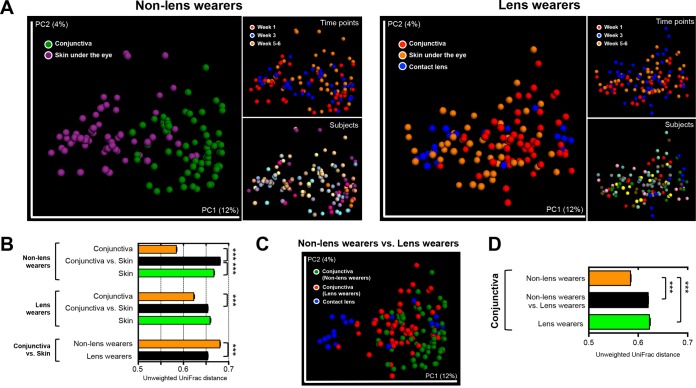
Beta diversity in conjunctiva, skin, and contact lens samples between non-lens wearers and lens wearers. Unweighted UniFrac distances were used to evaluate diversities between samples. (A) PCoA plot of bacterial communities by body site, time, and subject in non-lens wearers (left) and lens wearers (right). PC1, first principal component; PC2, second principal component. (B) Box plots of intragroup and intergroup distances in bacterial communities between conjunctiva and skin under the eye. (C) PCoA plot of ocular microbiota in non-lens wearers and lens wearers with bacterial communities of contact lenses. (D) Box plots of intragroup and intergroup distances in the ocular microbiota of non-lens wearers and lens wearers. ***, *P* value = <0.001.

**FIG 3:**
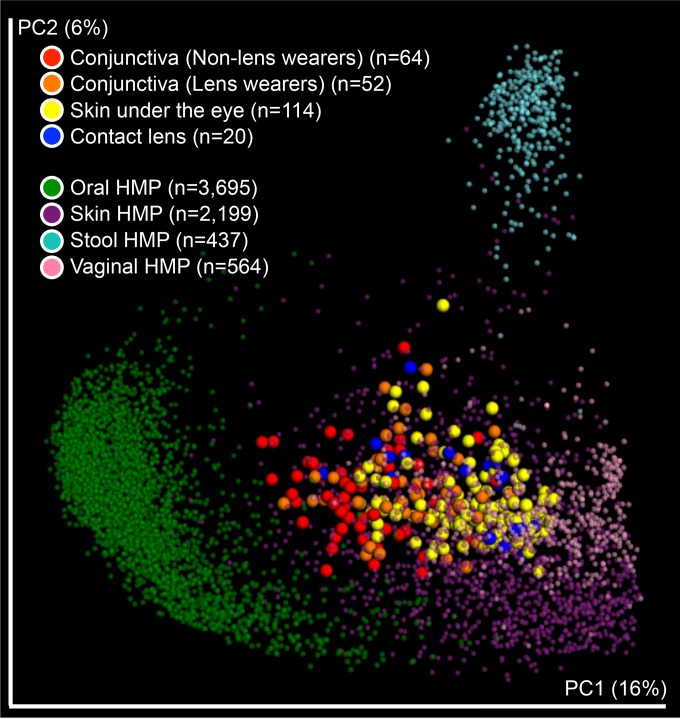
Beta diversity in conjunctiva, skin, and contact lens samples with samples from the HMP database. A PCoA plot was generated using unweighted UniFrac distances. Small dots (green, purple, cyan, and pink) indicate samples from the HMP database, and large dots (red, orange, yellow, and blue) indicate samples from this study.

There were no significant differences in bacterial alpha diversity between the conjunctiva of lens wearers and that of non-lens wearers (nonparametric Student’s *t* test; Fig. 1). The microbial structures of the conjunctiva and skin under the eye were more dissimilar in non-lens wearers than in lens wearers (unweighted UniFrac, nonparametric *t* test *P* value = <0.001; Fig. 2A and B). Consistently, the conjunctival microbiota of lens wearers was more similar to the microbiota of the skin under the eye than was the case with the non-lens wearers (unweighted UniFrac distance, *P* value = <0.001) (Fig. 2A and B; see also Fig. S4 in the supplemental material). However, there was no clustering by subject or sampling time points (Fig. 2A and B). Moreover, the conjunctival microbiota in lens wearers was more similar to human skin microbiota from a previous study (15) than to that in non-lens wearers (Fig. 3). Weighted UniFrac distances depicted on principal coordinate analysis (PCoA) plots also supported these results (see Fig. S4). Lens wearers also had higher interindividual variability in their ocular microbiota than non-lens wearers (unweighted UniFrac, nonparametric *P* value = <0.001; Fig. 2C and D).

**FIG 4:**
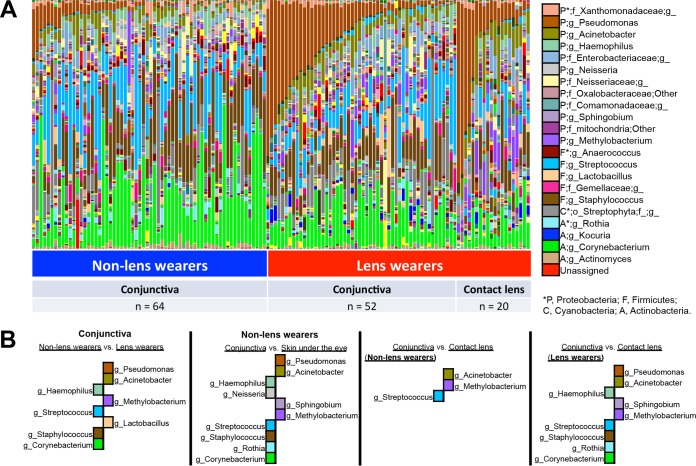
Differences in relative abundances of phylotypes in ocular microbiota between non-lens wearers and lens wearers. Each phylotype (>1% of average relative abundance in groups) is indicated by a different color at the genus level. (A) Bacterial taxon plots at the genus level. (B) Histogram of unique biomarker bacteria in each group. The LDA effect size (>3.0-fold) was used to detect unique biomarkers.

The conjunctival microbiota of non-lens wearers was influenced by gender (PERMANOVA *P* < 0.001; see Fig. S5A and B in the supplemental material). Numbers of *Acinetobacter* organisms and of members of family *Enterobacteriaceae* were increased and those of *Anaerococcus* were depleted in the ocular microbiota of female subjects compared to male subjects (LDA score = >3.0; see Fig. S5C and D). Regardless of gender, lens wearers had a skin-like conjunctival microbiota compared to non-lens wearers (see Fig. S5E and F). Compared to levels seen with non-lens wearers, the ocular microbiota of lens wearers was enriched in *Pseudomonas*, *Acinetobacter*, *Methylobacterium*, and *Lactobacillus* (LDA score = >3.0; Fig. 4). In non-lens wearers, these were detected at a higher relative abundance in skin samples than in the conjunctiva (except for *Lactobacillus*) (Fig. 4), suggesting that these bacteria could be classified as skin bacteria. Levels of *Haemophilus*, *Streptococcus*, *Staphylococcus*, and *Corynebacterium* were depleted in the ocular microbiota of lens wearers compared to non-lens wearers (LDA score = >3.0; Fig. 4). Contact lenses demonstrated a higher relative abundance of *Acinetobacter* and *Methylobacterium* than the conjunctiva (LDA score = >3.0; Fig. 4).

We compared the conjunctival microbiota with that in the skin under the eye. In relation to the skin, the conjunctiva of normal eye (non-lens wearers) had higher abundances of *Haemophilus*, *Neisseria*, *Streptococcus*, *Staphylococcus*, *Rothia*, and *Corynebacterium* and lower abundances of *Pseudomonas*, *Acinetobacter*, *Sphingobium*, and *Methylobacterium* (LDA score = >3.0; Fig. 4B). However, regardless of wearing or not wearing lenses, SourceTracker analyses using the V4 region from data generated in a previous study of the skin microbiome (16) showed that the conjunctival microbiota of both lens wearers and non-lens wearers had higher proportions of hand-like bacteria than of face-like bacteria (*P* < 0.0001; see Fig. S3B in the supplemental material).

There were no significant differences in bacterial diversity and composition between the conjunctival microbiota and skin microbiota at different time points (see Fig. S6 in the supplemental material).

Predictive functional profiling showed that the conjunctiva of lens wearers had a higher proportion of bacterial genes related to cell motility and xenobiotic biodegradation than the conjunctiva of non-lens wearers. On the other hand, the conjunctival microbiota of non-wearers had higher relative content of genes related to genetic processing and nucleotide metabolism (see Fig. S7 in the supplemental material).

## DISCUSSION

Wearing contact lenses is a known risk factor for development of microbial keratitis and other inflammatory eye conditions (17, 18). Several researchers have tried to identify the causative organism of these contact lens-related diseases using culture-dependent methods (1, 18). To the best of our knowledge, this was a pioneering study in identifying the structure of the microbiota from contact lenses and conjunctiva of contact lenses wearers using 16S rRNA gene sequencing. While mostly illustrated by results of studies of the gut, the concept of colonization resistance, a protective effect shown by microbiota against invasions of foreign bacteria, has been suggested for the eye (7). This would imply that some impacts on the eye microbiota, such as those related to wearing contacts, might decrease the resilience or self-restoring capacity of the ecosystem. Although the data represent only 3 time points, we found relatively high stability of the eye microbiome. The healthy eye is constantly wetted by tears, which may provide a constant chemical environment.

The bacterial diversity of the conjunctiva is higher than that in the skin. Moreover, conjunctiva had an alpha diversity similar to that of the oral microbiota (see Fig. S3 in the supplemental material). These results are remarkable considering the antimicrobial effects of tear compounds (19).

This study showed that the eye microbiota of lens wearers is different from that of non-wearers, resembling more closely the microbiota of the skin. The results are consistent with those previously found with culture-dependent methods (20–22). Consistently, the presence of *Staphylococcus*, *Propionibacterium*, *Corynebacterium*, *Bacillus*, *Micrococcus*, *Rothia* (previously assigned to *Stomatococcus*), and *Pseudomonas* in cultures from contact lenses has been reported (1). We also detected members of other taxa in contact lenses, such as *Streptococcus*, *Methylobacterium*, and *Acinetobacter*, and members of families *Oxalobacteraceae* and *Enterobacteriaceae* at a relative abundance of >1% in more than half of the samples. In addition, *Streptophyta* spp. (chloroplast DNA, possibly an environmental contaminant) showed a nonsignificant tendency (nonparametric *t* test; *P* = 0.26) to be higher in the conjunctiva of lens wearers than in that of non-lens wearers (Fig. 4; see also Fig. S5 in the supplemental material). We would not have detected differences in the conjunctival microbiota between lens wearers and non-lens wearers in this study if we had included samples collected from the ocular surface after the use of topical proparacaine hydrochloride anesthetic, highlighting the importance of sampling microbiome sites with minimal perturbations. The anesthetic may have diluted or washed away bacteria from the ocular surface.

By comparing the microbiota of ocular conjunctiva to that of skin under the eye, we found that normal *Corynebacterium* and *Staphylococcus* skin bacteria were detected at higher relative abundance in the eye than in the skin, consistent with a recent study showing the distribution of skin microbiome by body sites (16). Tear lactoferrin plays a role in reducing levels of *Staphylococcus epidermidis* biofilms (23). However, in this study, we found that *Staphylococcus* levels were lower in lens wearers than in non-lens wearers. There are not many reports of studies using molecular methods in the diseased eye. Lee et al. (6) studied blepharitis using different sampling sites (eyelashes and tears), sequencing regions, and platforms (V1 to V3 16S rRNA genes with Roche-454). Their results showed that blepharitis patients (*n* = 7) had increased *Staphylococcus*, *Streptophyta*, *Corynebacterium*, and *Enhydrobacter* levels in their eyelashes and tears in relation to controls (*n* = 4), none of which were increased in our lens wearers. In addition to the different sites sampled, their patients were older than ours (59 [± 16.6]-year-old patients in the study by Lee et al. and 26 [± 4.5]-year-old patients in our study). More studies are needed to understand which are the opportunistic pathogens associated with infections in lens wearers. Other skin bacteria were enriched in contact lenses and in the conjunctiva of lens wearers, which have increased relative abundances of *Pseudomonas*, *Acinetobacter*, and *Methylobacterium.* Representatives of these genera are considered to be opportunistic pathogens in conjunctivitis, keratitis, and endophthalmitis (24–26). Enrichment of skin bacteria caused by wearing contact lenses suggests that contact lenses could function as a medium to transfer skin bacteria to the ocular surface. Alternatively, contact lenses may be exerting selective pressure on ocular bacterial communities in favor of skin-like bacteria.

Given that people use their fingers (no matter how well washed) to put in contact lenses, we tested whether conjunctival bacterial OTUs in lens wearers originate from hands, and results suggested that they do not. Consistently, evidence suggests that transplantation of microbiota between body sites changes the bacterial population structure only temporarily (27). An experiment in which lens wearers use or do not use sterile gloves to insert their lenses would clarify the origin of the bacteria in contact lenses.

The different taxa in the conjunctiva of lens wearers are reflected in the predicted bacterial gene content, so the differences are not redundant in gene content. We could reliably predict bacterial gene profiles (mean accuracy =0.99 ± 0.03 standard deviation [SD] for core/bacterial housekeeping functions; minimum accuracy = 0.82 for membrane-associated functions) (28); further metagenomic/transcriptomic research is needed to elucidate whether bacterial gene functions confer reduced protection against ocular infections.

Previous studies have suggested that the commensal microbiota of the ocular surface could interact with the host in immune system to suppress microbial pathogenicity (4, 6, 7), and the impact of wearing contact lenses may affect this protective function. Our report provides novel insights into possible mechanisms by which wearing contact lenses increases eye infection risks. Further research is required to determine if the risk is related to contaminating the lenses with bacteria from the skin of the finger or if contact lenses exert selective pressures on the eye bacterial community in favor of skin bacteria.

## MATERIALS AND METHODS

### Sample collection.

The study was performed under IRB protocol S12-03905 of the NYU Medical Center. Subjects seeking routine eye care at the ophthalmology practice at the NYU Medical Center from October 2013 to June 2014 were sampled from their left and right eyes. A total of 58 subjects (40 females and 18 males) provided samples; 20 of those subjects (including 9 lens wearers and 11 non-lens wearers) were followed longitudinally, with biweekly samples collected for 6 weeks (see Table S1 and Table S2 in the supplemental material). Dry sterile cotton swabs (Fisher Scientific) used to swab sampling sites were immediately placed in sterile cryogenic tubes (Thermo Scientific). Sites sampled included right and left conjunctivas, skin under the right and left eyes, and contact lens (250 total samples, including 116 conjunctiva samples, 114 skin samples, and 20 contact lens samples). Samples were immediately transported to the laboratory on ice and frozen at − 80°C. A total of 38 additional subjects were sampled once (including 16 lens wearers; *n* = 75) (see Table S3) in an ophthalmology office after utilization of a 50-μl drop of topical 0.5% proparacaine hydrochloride anesthetic. Negative-control swabs (*n* = 3, with no sample) were also included.

### DNA extraction and sequencing.

DNA extraction was carried out using MoBio (CA, USA) PowerSoil-htp 96 well soil DNA isolation plates according to the instructions provided by the manufacturer. Extracted DNA from samples was stored at −20°C until sequencing. The V4 region of 16s rRNA gene was amplified by PCR using barcoded primers, as previously described (29). To rule out and control for possible reagent contamination, reagents for DNA extraction and for PCR amplification were also sequenced as controls (30). The amplicons were then pooled in equimolar ratios and purified using a QIAquick PCR purification kit (Qiagen Inc., CA, USA). The pooled amplicons were sequenced on an Illumina MiSeq platform (Genome Technology Center of NYU Medical Center, NY) using a paired-end technique (2 150-cycle runs).

### Data analysis.

The 16S rRNA sequence analyses were performed with the QIIME suite of software tools (v1.8) (31). The filtered sequence reads (Phred ≥ Q20) were used to pick the operational taxonomic units (OTUs), with an open-reference OTU picking method based on 97% identity to entries in the Greengenes database (v13_8). Negative-control-derived OTUs were discarded from the OTU table using a filtration script (filter_otus_from_otu_table.py) in QIIME. After the chimeric sequences were removed using UCHIME (32), all communities were rarefied to 2,090 reads per sample. For comparison of levels of beta diversity between communities, the unweighted/weighted UniFrac distances (33) were calculated and PERMANOVA (34) was used to test significance. Linear discriminant analysis effect size (LEfSe) (35) was used to detect unique biomarkers by determinations of the relative abundances of the members of the bacterial taxonomies. Predictive functional analysis was performed using PICRUSt (28) with Kegg Orthology (KO) classification (36).

To determine the overlap between OTUs of the eye samples and OTUs found in other skin sites, we used SourceTracker (37) with a previously reported 16S rRNA gene data set derived from samples from human face and hand (16).

The HMP data set (15) of 16S rRNA (regions V3 to V5) sequences was downloaded from the NIH HMP website (hmpdacc.org) and was trimmed to contain only the V4 region of the 16S rRNA gene using BioPerl (http://www.bioperl.org/wiki/Main_Page). The 16S rRNA (V4 region) sequences from the skin of the face (16) were downloaded from EMBL EBI database (ERP005182). The sequences from the Bouslimani study and the V4-trimmed HMP data set were merged with sequences from this study. The QIIME suite (v1.8) was used to pick OTUs from the merged sequences using the closed-reference method. Then, all communities were rarefied to 1,000 sequences per sample to calculate bacterial diversity.

### Nucleotide sequence accession number.

The raw sequences supporting the results of this article are available in the European Nucleotide Archive repository under accession no. PRJEB12498 (http://www.ebi.ac.uk/ena/data/view/PRJEB12498).

## SUPPLEMENTAL MATERIAL

Figure S1  Bacterial communities of the conjunctiva classified by sampling locations. (A) Rarefaction plots of ocular microbiota classified by sampling locations using phylogenetic diversity (PD) whole-tree matrix data (left) and numbers of observed species (right). Alpha diversity was generated with rarefication to 2,090 reads per sample. The nonparametric *P* values were calculated using 999 Monte Carlo permutations. ***, *P* value = <0.001. (B) Taxon bar plots depicting bacterial structure classified by sampling sites at the phylum (left) and genus (right) levels. Each phylotype (>1% of average relative abundance in groups) is indicated by a different color. The nonparametric *P* values were calculated using 999 Monte Carlo permutations. *, *P* value = <0.05. (C) Beta diversity of conjunctival bacterial communities classified by sampling locations. Unweighted (left) and weighted (right) UniFrac distances were plotted using PCoA. The significant differences in PCoA plots were analyzed using PERMANOVA. Box plots show intragroup distances of ocular bacterial communities (middle). The nonparametric *P* values were calculated using 999 Monte Carlo permutations. ***, *P* value = <0.001. Download Figure S1, PDF file, 0.4 MB

Figure S2  Bacterial communities of the conjunctiva collected at the ophthalmology practice. (A) Rarefaction plots of ocular microbiota using PD whole-tree matrix data (left) and numbers of observed species (right). Alpha diversity was generated with rarefication to 2,090 reads per sample. The nonparametric *P* values were calculated using 999 Monte Carlo permutations. (B) PCoA plots of ocular bacterial communities using unweighted (left) and weighted (right) UniFrac distances. The significant differences in PCoA plots were obtained using PERMANOVA. (C) Each phylotype (>1% of average relative abundance in groups) is indicated by a different color at the genus level. The nonparametric *P* values (<0.05) calculated using 999 Monte Carlo permutations and LDA effect size (>3.0-fold) were used to detect unique biomarkers. *, *P* value = <0.05. Download Figure S2, PDF file, 1.5 MB

Figure S3  Comparison with previous datasets. (A) Rarefaction plots of bacterial communities in the conjunctiva, skin, and contact lens samples with HMP database and the other skin study. The number of observed species was used to evaluate alpha diversity. (B) Source proportions for conjunctiva and contact lens determined using SourceTracker. The average contributions of skin sites (hand and face) to the bacterial communities of conjunctiva or contact lens were predicted by SourceTracker. ***, *P* value = <0.0001. Download Figure S3, PDF file, 0.1 MB

Figure S4  Beta diversity in the conjunctiva, skin, and contact lens samples between non-lens wearers and lens wearers determined using UniFrac distances. (A and B) Comparison of intergroup unweighted (A) and weighted (B) UniFrac distances. (C and D) Statistical significance of the unweighted (C) and weighted (D) beta diversity differences determined using PERMANOVA. (E) Box plots of intragroup distances of ocular bacterial communities. Nonparametric *P* values were calculated using 999 Monte Carlo permutations. ***, *P* value = <0.001. (F to H) PCoA plot with weighted UniFrac distances showing non-lens wearers (F), lens wearers (G), and all groups (H). Download Figure S4, PDF file, 0.4 MB

Figure S5  Bacterial diversity in the conjunctiva, skin, and contact lens samples by gender. Unweighted UniFrac distances were used to evaluate diversities between samples. (A and B) PCoA plots of bacterial communities in conjunctiva and skin under the eye of non-lens wearers (A) or lens wearers (B). The significant differences in PCoA plots were obtained using PERMANOVA. (C and D) Histogram of unique biomarker conjunctival bacteria in each gender of non-lens wearers (C) or lens wearers (D). The LDA effect size (>3.0-fold) was used to detect unique biomarkers. (E) PCoA plot of bacterial communities classified by lens wear and gender. (F) Box plots of intergroup distances in bacterial communities between conjunctiva and skin under the eye. Download Figure S5, PDF file, 1.3 MB

Figure S6  Bacterial communities by time point, in each body site, and in contact lenses. (A and B) Rarefaction plots by time point, using PD whole-tree matrix data (A) and numbers of observed species (B). Nonparametric *P* values (<0.05) were calculated using 999 Monte Carlo permutations. **, *P* value = <0.05. (C) Taxon bar plots depicting bacterial structure by time point. Each phylotype (>1% of average relative abundance in groups) is indicated by a different color at the genus level. Download Figure S6, PDF file, 0.7 MB

Figure S7  Differences in predictive functional profiling of the ocular microbiome between lens wearers and non-lens wearers. The predictive functional profile was constructed using PICRUSt from the bacterial composition information derived by the 16S rRNA gene-based sequencing technique used here. LDA effect size (>3.0-fold) was used to detect significant differences between groups. Download Figure S7, PDF file, 0.1 MB

Table S1  The summary of analyzed-sequence information. Table S1, PDF file, 0.1 MB

Table S2  The summary of analyzed-sequence information with samples collected at the laboratory. Table S2, PDF file, 0.1 MB

Table S3  The summary of analyzed-sequence information with samples collected at the ophthalmology practice. Table S3, PDF file, 0.1 MB
