# Supplementary figures and images for: Changes in the Eye Microbiota Associated with Contact Lens Wearing
Source: mBio. 2016 Mar 22;7(2):e00198-16. doi: 10.1128/mBio.00198-16 (PMC4817251; doi:10.1128/mBio.00198-16)

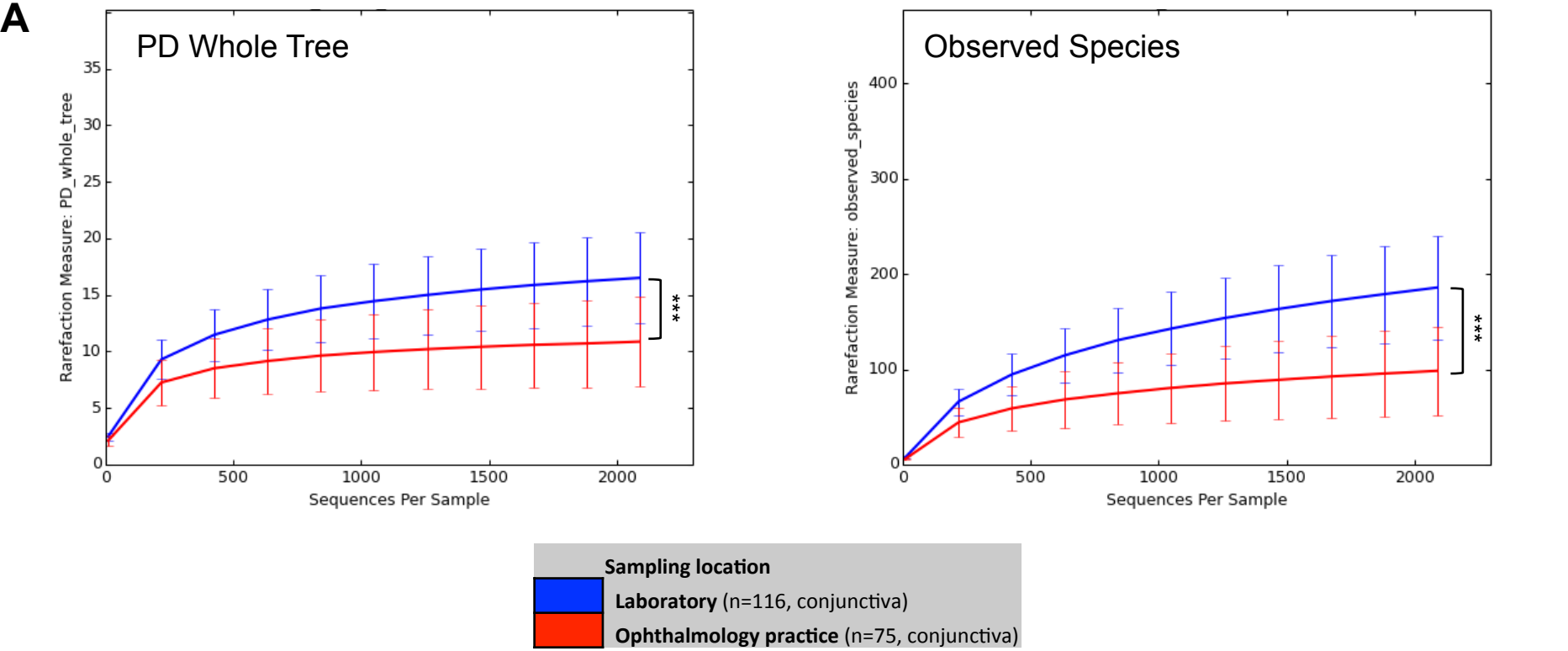

Supplement: Figure S1 — Bacterial communities of the conjunctiva classified by sampling locations. (A) Rarefaction plots of ocular microbiota classified by sampling locations using phylogenetic diversity (PD) whole-tree matrix data (left) and numbers of observed species (right). Alpha diversity was generated with rarefication to 2,090 reads per sample. The nonparametric P values were calculated using 999 Monte Carlo permutations. ***, P value = <0.001. (B) Taxon bar plots depicting bacterial structure classified by sampling sites at the phylum (left) and genus (right) levels. Each phylotype (>1% of average relative abundance in groups) is indicated by a different color. The nonparametric P values were calculated using 999 Monte Carlo permutations. *, P value = <0.05. (C) Beta diversity of conjunctival bacterial communities classified by sampling locations. Unweighted (left) and weighted (right) UniFrac distances were plotted using PCoA. The significant differences in PCoA plots were analyzed using PERMANOVA. Box plots show intragroup distances of ocular bacterial communities (middle). The nonparametric P values were calculated using 999 Monte Carlo permutations. ***, P value = <0.001. Download [file mbo002162742sf1.pdf]

A

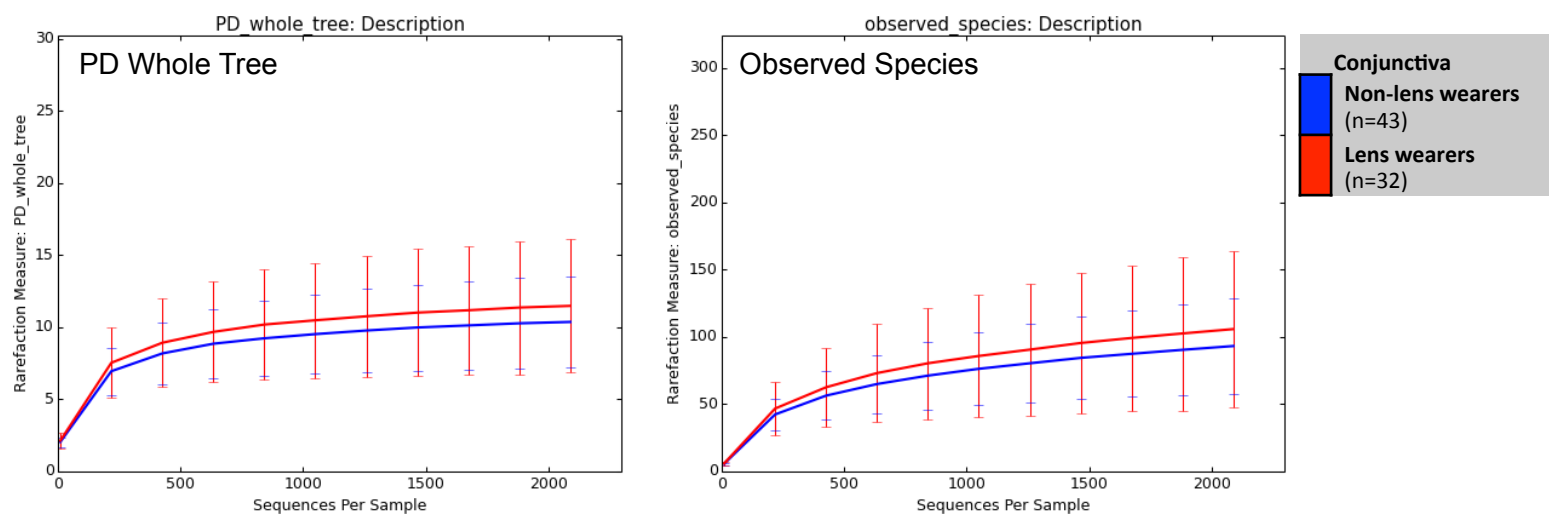

B

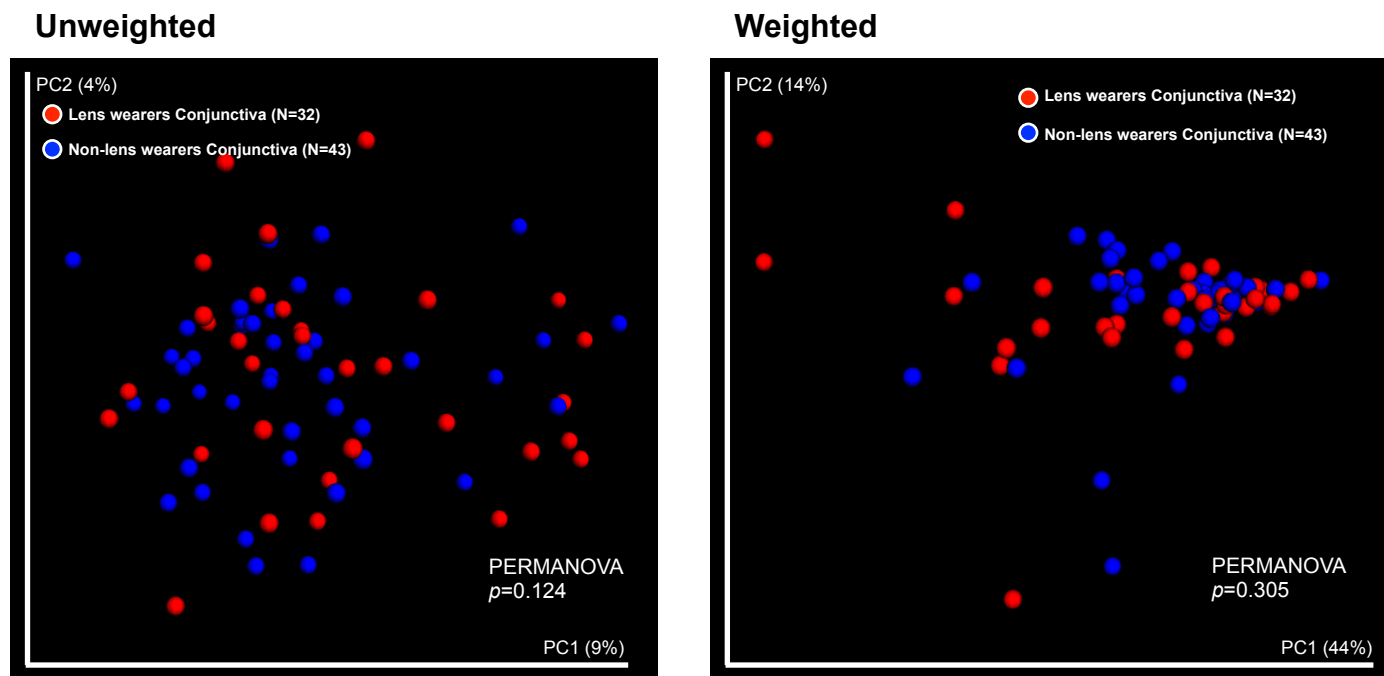

C

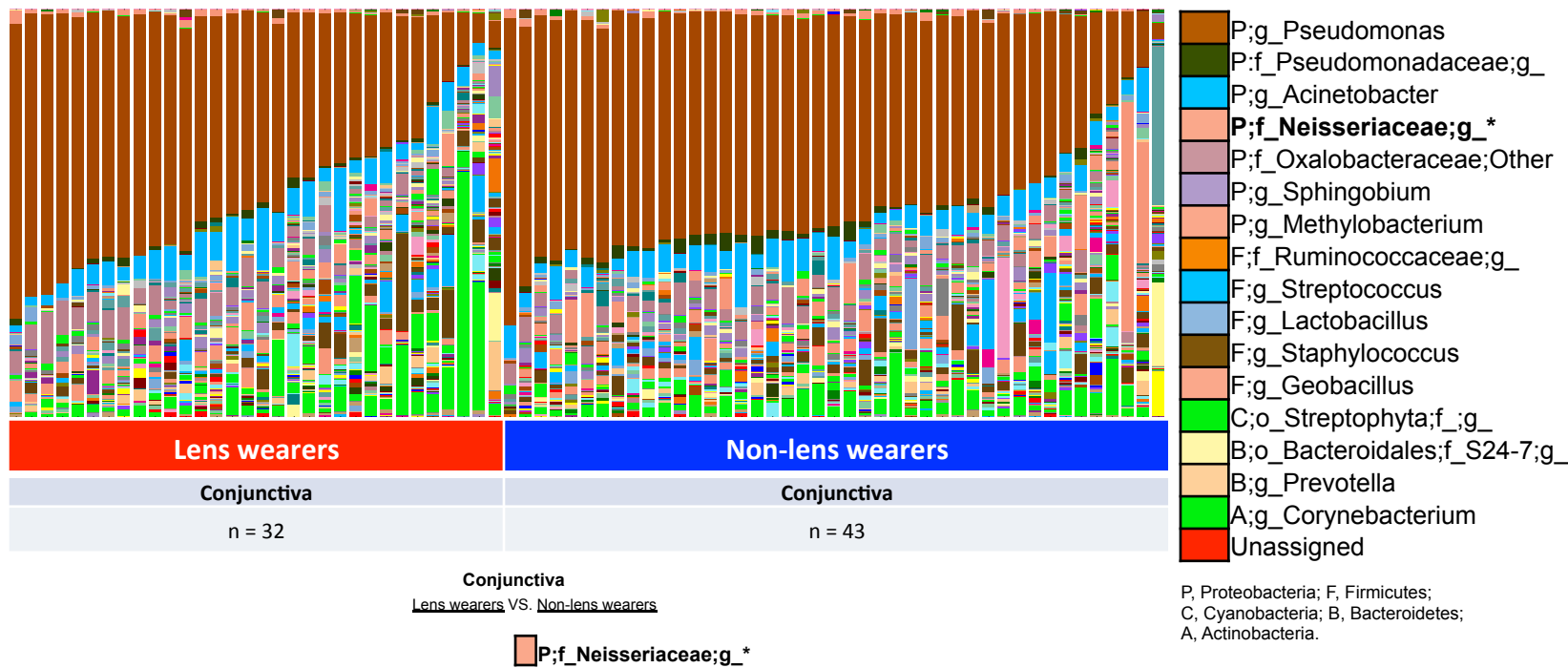

Supplement: Figure S2 — Bacterial communities of the conjunctiva collected at the ophthalmology practice. (A) Rarefaction plots of ocular microbiota using PD whole-tree matrix data (left) and numbers of observed species (right). Alpha diversity was generated with rarefication to 2,090 reads per sample. The nonparametric P values were calculated using 999 Monte Carlo permutations. (B) PCoA plots of ocular bacterial communities using unweighted (left) and weighted (right) UniFrac distances. The significant differences in PCoA plots were obtained using PERMANOVA. (C) Each phylotype (>1% of average relative abundance in groups) is indicated by a different color at the genus level. The nonparametric P values (<0.05) calculated using 999 Monte Carlo permutations and LDA effect size (>3.0-fold) were used to detect unique biomarkers. *, P value = <0.05. Download [file mbo002162742sf2.pdf]

**A**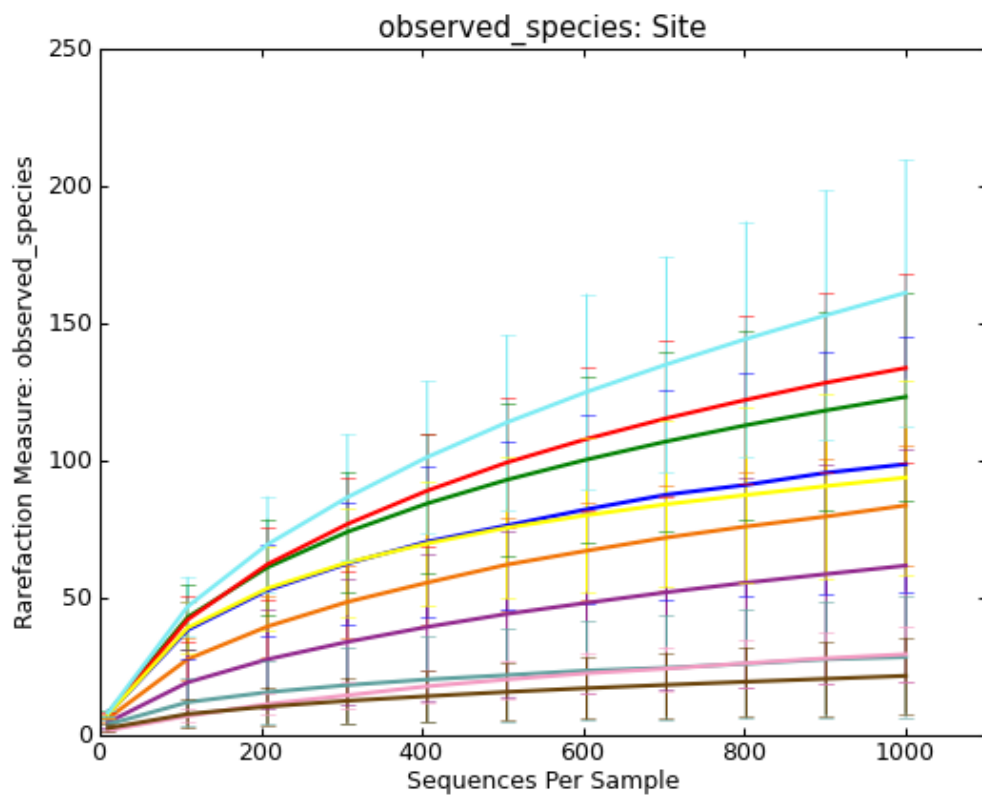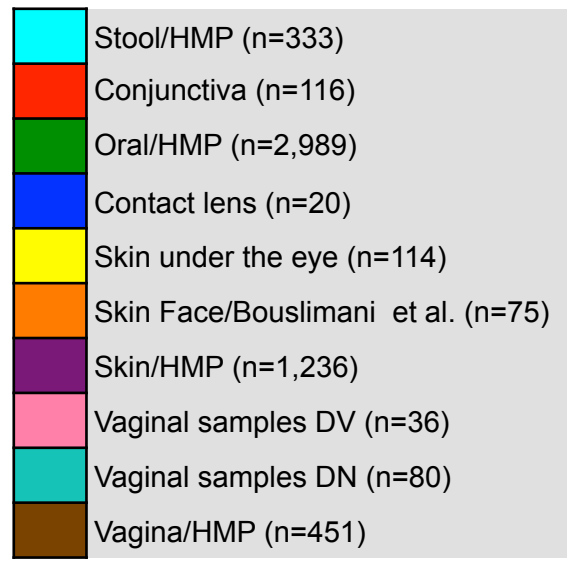**B**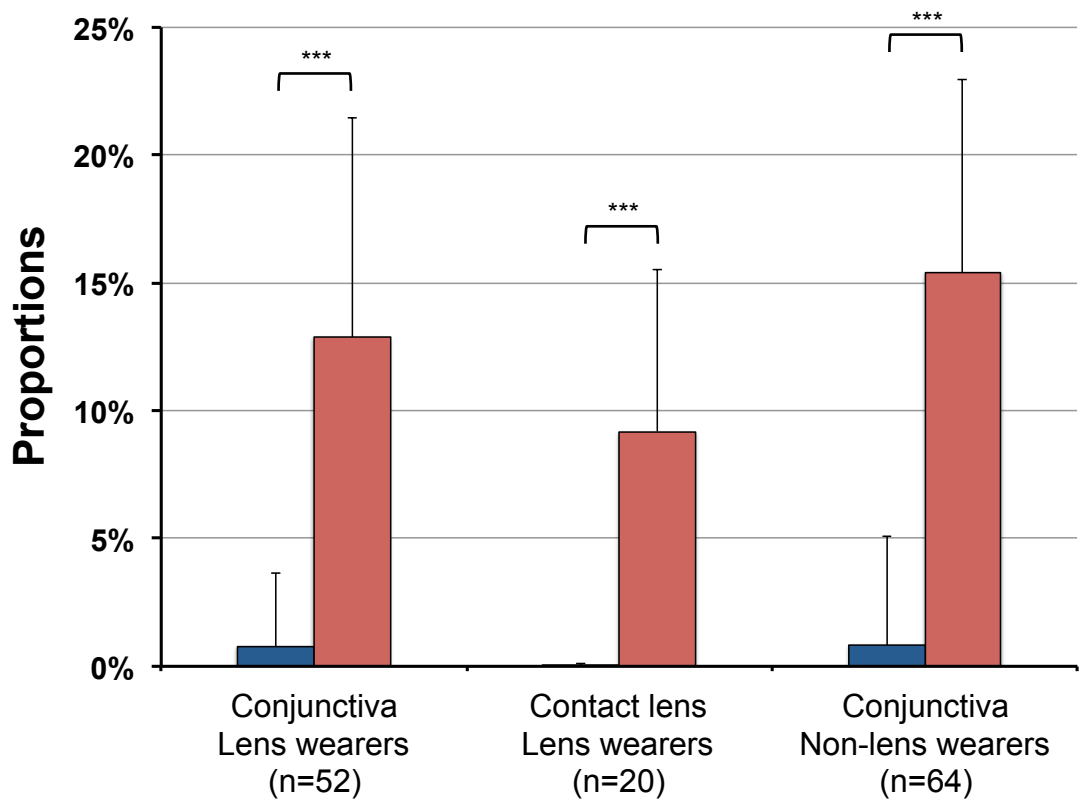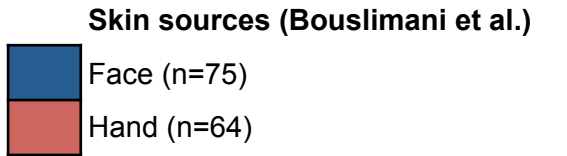

Supplement: Figure S3 — Comparison with previous datasets. (A) Rarefaction plots of bacterial communities in the conjunctiva, skin, and contact lens samples with HMP database and the other skin study. The number of observed species was used to evaluate alpha diversity. (B) Source proportions for conjunctiva and contact lens determined using SourceTracker. The average contributions of skin sites (hand and face) to the bacterial communities of conjunctiva or contact lens were predicted by SourceTracker. ***, P value = <0.0001. Download [file mbo002162742sf3.pdf]

# Conjunctiva

**Lens wearers VS. Non-lens wearers**

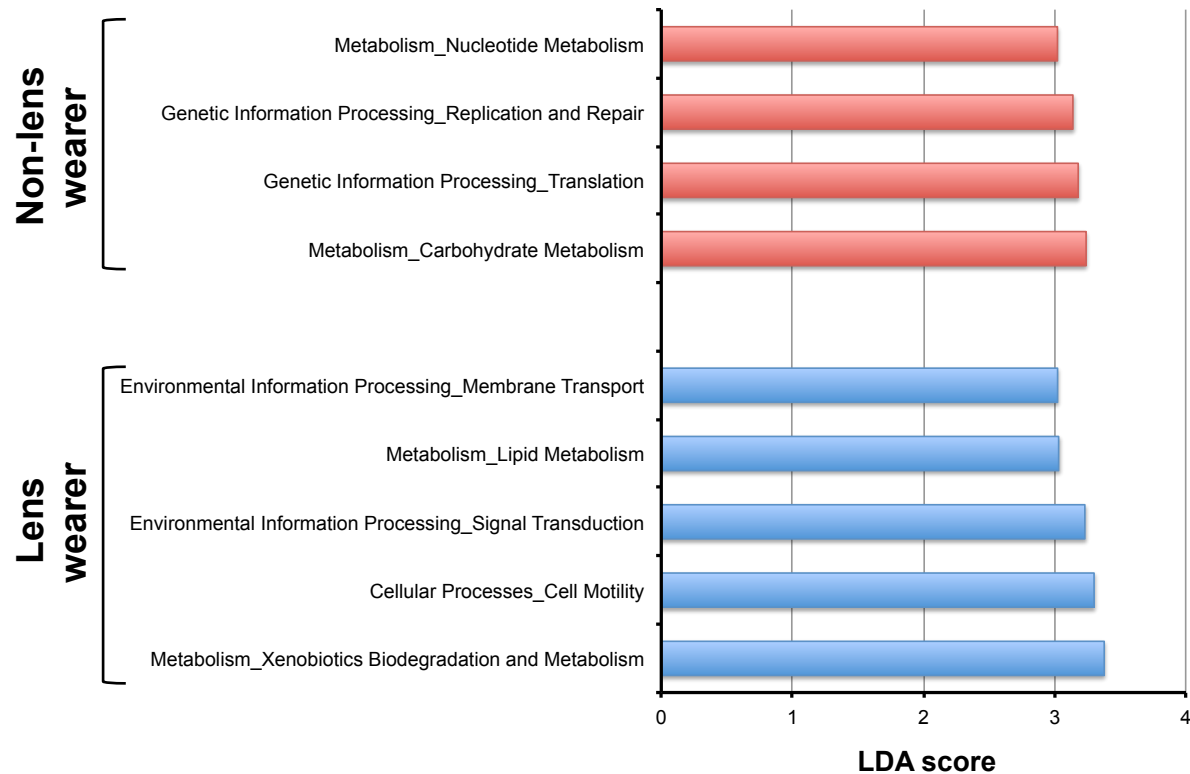

Supplement: Figure S7 — Differences in predictive functional profiling of the ocular microbiome between lens wearers and non-lens wearers. The predictive functional profile was constructed using PICRUSt from the bacterial composition information derived by the 16S rRNA gene-based sequencing technique used here. LDA effect size (>3.0-fold) was used to detect significant differences between groups. Download [file mbo002162742sf7.pdf]
