# Supplementary material for: Changes in the Eye Microbiota Associated with Contact Lens Wearing
Source: mBio. 2016 Mar 22;7(2):e00198-16. doi: 10.1128/mBio.00198-16 (PMC4817251; doi:10.1128/mBio.00198-16)
Supplement: Figure S4 — Beta diversity in the conjunctiva, skin, and contact lens samples between non-lens wearers and lens wearers determined using UniFrac distances. (A and B) Comparison of intergroup unweighted (A) and weighted (B) UniFrac distances. (C and D) Statistical significance of the unweighted (C) and weighted (D) beta diversity differences determined using PERMANOVA. (E) Box plots of intragroup distances of ocular bacterial communities. Nonparametric P values were calculated using 999 Monte Carlo permutations. ***, P value = <0.001. (F to H) PCoA plot with weighted UniFrac distances showing non-lens wearers (F), lens wearers (G), and all groups (H). Download [file mbo002162742sf4.pdf]

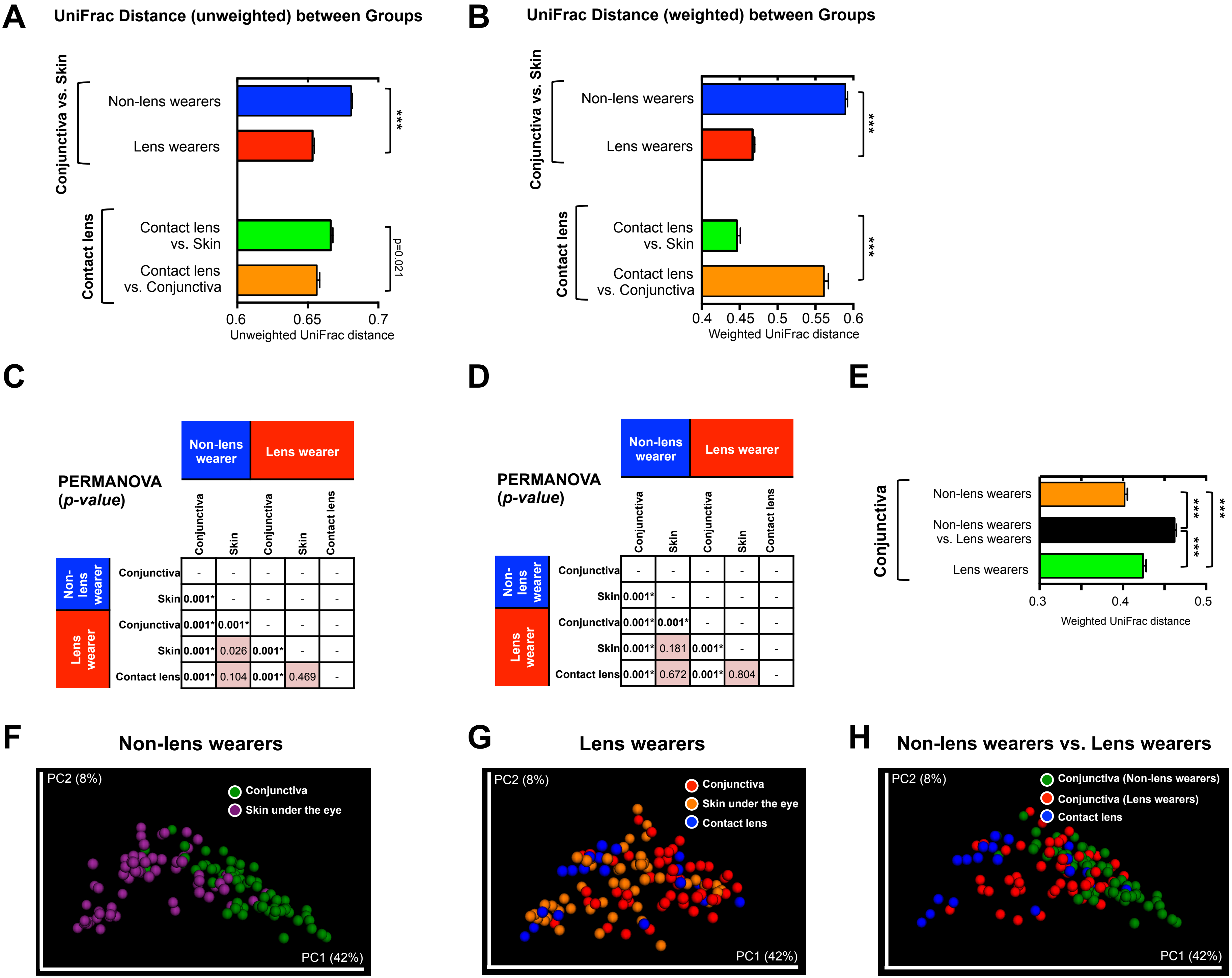

C PERMANOVA (*p-value*)

		Non-lens wearer: Conjunctiva	Non-lens wearer: Skin	Lens wearer: Conjunctiva	Lens wearer: Skin	Lens wearer: Contact lens
Non-lens wearer	Conjunctiva	-	-	-	-	-
	Skin	0.001*	-	-	-	-
Lens wearer	Conjunctiva	0.001*	0.001*	-	-	-
	Skin	0.001*	0.026	0.001*	-	-
	Contact lens	0.001*	0.104	0.001*	0.469	-

D PERMANOVA (*p-value*)

		Non-lens wearer: Conjunctiva	Non-lens wearer: Skin	Lens wearer: Conjunctiva	Lens wearer: Skin	Lens wearer: Contact lens
Non-lens wearer	Conjunctiva	-	-	-	-	-
	Skin	0.001*	-	-	-	-
Lens wearer	Conjunctiva	0.001*	0.001*	-	-	-
	Skin	0.001*	0.181	0.001*	-	-
	Contact lens	0.001*	0.672	0.001*	0.804	-
