# Supplementary material for: Changes in the Eye Microbiota Associated with Contact Lens Wearing
Source: mBio. 2016 Mar 22;7(2):e00198-16. doi: 10.1128/mBio.00198-16 (PMC4817251; doi:10.1128/mBio.00198-16)
Supplement: Figure S6 — Bacterial communities by time point, in each body site, and in contact lenses. (A and B) Rarefaction plots by time point, using PD whole-tree matrix data (A) and numbers of observed species (B). Nonparametric P values (<0.05) were calculated using 999 Monte Carlo permutations. **, P value = <0.05. (C) Taxon bar plots depicting bacterial structure by time point. Each phylotype (>1% of average relative abundance in groups) is indicated by a different color at the genus level. Download [file mbo002162742sf6.pdf]

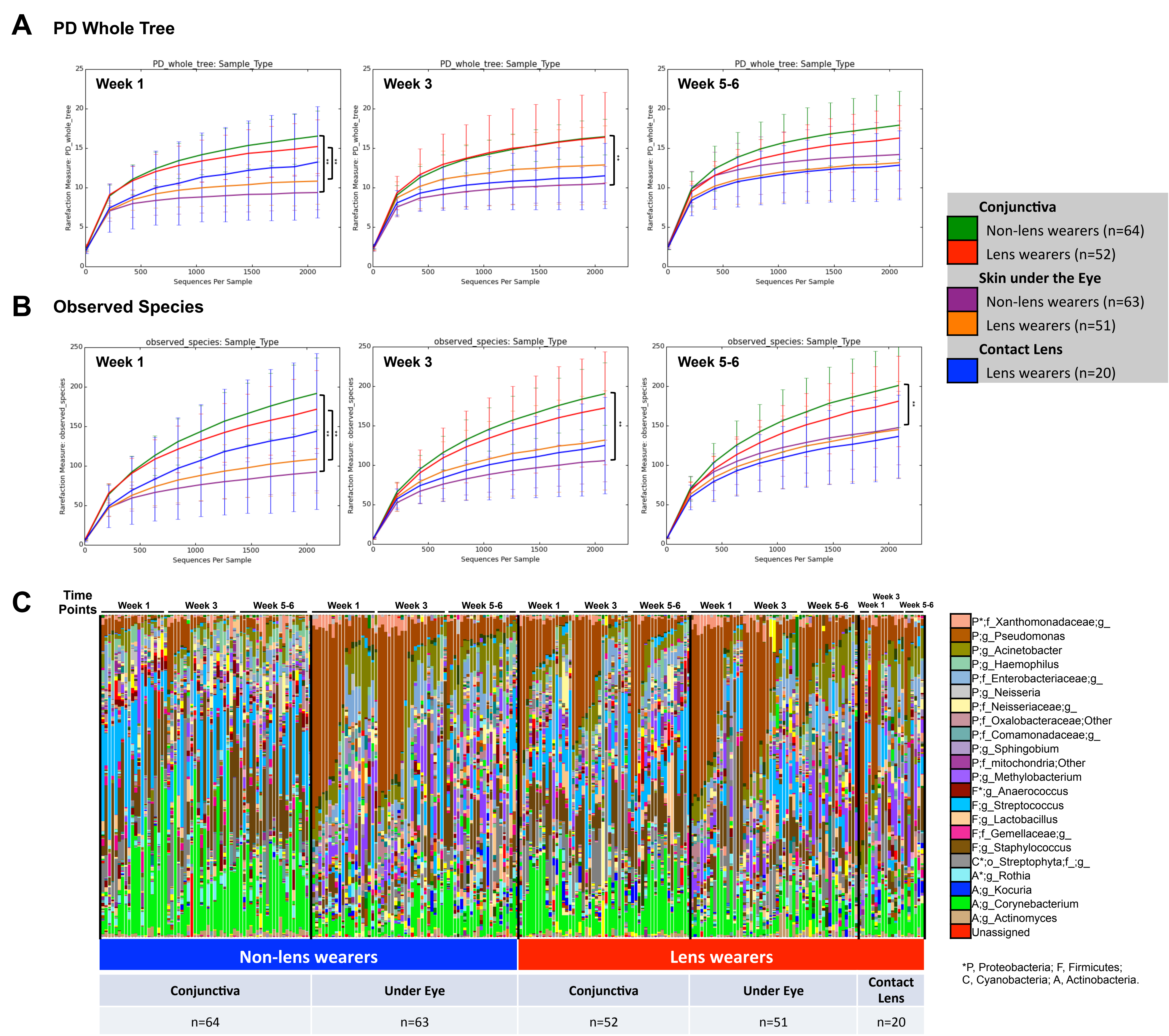
A
PD Whole Tree
PD_whole_tree: Sample_Type
Week 1
Week 3
Week 5-6
Rarefaction Measure: PD_whole_tree
Sequences Per Sample
**
B
Observed Species
observed_species: Sample_Type
Rarefaction Measure: observed_species
Conjunctiva
Non-lens wearers (n=64)
Lens wearers (n=52)
Skin under the Eye
Non-lens wearers (n=63)
Lens wearers (n=51)
Contact Lens
Lens wearers (n=20)
C
Time Points
Week 1
Week 3
Week 5-6
P*;f_Xanthomonadaceae;g_
P;g_Pseudomonas
P;g_Acinetobacter
P;g_Haemophilus
P;f_Enterobacteriaceae;g_
P;g_Neisseria
P;f_Neisseriaceae;g_
P;f_Oxalobacteraceae;Other
P;f_Comamonadaceae;g_
P;g_Sphingobium
P;f_mitochondria;Other
P;g_Methylobacterium
F*;g_Anaerococcus
F;g_Streptococcus
F;g_Lactobacillus
F;f_Gemellaceae;g_
F;g_Staphylococcus
C*;o_Streptophyta;f_;g_
A*;g_Rothia
A;g_Kocuria
A;g_Corynebacterium
A;g_Actinomyces
Unassigned
Non-lens wearers
Lens wearers
Conjunctiva
Under Eye
Contact Lens
n=64
n=63
n=52
n=51
n=20
*P, Proteobacteria; F, Firmicutes; C, Cyanobacteria; A, Actinobacteria.
