# Supplementary material for: Changes in the Eye Microbiota Associated with Contact Lens Wearing
Source: mBio. 2016 Mar 22;7(2):e00198-16. doi: 10.1128/mBio.00198-16 (PMC4817251; doi:10.1128/mBio.00198-16)
Supplement: Table S1 — The summary of analyzed-sequence information. [file mbo002162742st1.pdf]

Table S1. The summary of analyzed sequences information.

**Wearers Type**	**Non-lens wearers (n=30)**		**Lens wearers (n=25)**			**Negative control**	**Total**
**Sample Type**	**Conjunctiva**	**Skin under eye**	**Conjunctiva**	**Skin under eye**	**Contact lens**		
No. of samples	107	63	84	51	20	5	325
**Total no. of Seqs**	**2,977,673**	**931,891**	**2,082,499**	**861,323**	**327,353**	**53**	**7,180,739**
Mean no. of Seqs (± Std dev)	27,828 ± 12,887	14,791 ± 11,485	24,791 ± 14,395	16,888 ± 10,640	16,367 ± 10,790	11 ± 11	22,094 ± 13,713
**Total no. of Seqs yielding OTUs**	**2,920,899**	**902,685**	**2,031,230**	**835,398**	**319,884**	**56**	**7,010,096 (2.4% loss)**
Mean no. of Seqs yielding OTUs (± Std dev)	27,298 ± 12,742	14,328 ± 11,222	24,181 ± 13,973	16,380 ± 10,466	15,994 ± 10,743	11 ± 11	21,569 ± 13,472
**No. of observed OTU types**	**7,392**	**4,064**	**6,849**	**2,300**	**4,069**	**30**	**11,750**
**Mean no. of OTU types** (± Std dev)	460 ± 157	207 ± 106	380 ± 184	240 ± 137	260 ± 183	7 ± 6	319 ± 182
