# Supplementary material for: Changes in the Eye Microbiota Associated with Contact Lens Wearing
Source: mBio. 2016 Mar 22;7(2):e00198-16. doi: 10.1128/mBio.00198-16 (PMC4817251; doi:10.1128/mBio.00198-16)
Supplement: Table S2 — The summary of analyzed-sequence information with samples collected at the laboratory. [file mbo002162742st2.pdf]

Table S2. The summary of analyzed sequences information with samples collected at laboratory.

Sampling Site	Laboratory					Total
Wearers Type	Non-lens wearers (n=11)		Lens wearers (n=9)			
Sample Type	Conjunctiva	Skin under eye	Conjunctiva	Skin under eye	Contact lens	
No. of samples	64	63	52	51	20	250
**Total no. of Seqs**	**1,987,887**	**931,891**	**1,311,429**	**861,323**	**327,353**	**5,419,883**
Mean no. of Seqs (± Std dev)	31,061 ± 12,319	14,791 ± 11,485	25,220 ± 12,913	16,888 ± 10,640	16,367 ± 10,790	21,680 ± 13,536
**Total no. of Seqs yielding OTUs**	**1,962,312**	**902,685**	**1,287,614**	**835,398**	**319,884**	**5,307,893 (2.1% loss)**
Mean no. of Seqs yielding OTUs (± Std dev)	30,661 ± 12,160	14,328 ± 11,222	24762 ± 12700	16,380 ± 10,466	15,994 ± 10,743	21,232 ± 13,382
**No. of observed OTU types**	**6,086**	**4,064**	**5,632**	**2,300**	**4,069**	**10,728**
