# Supplementary material for: Changes in the Eye Microbiota Associated with Contact Lens Wearing
Source: mBio. 2016 Mar 22;7(2):e00198-16. doi: 10.1128/mBio.00198-16 (PMC4817251; doi:10.1128/mBio.00198-16)
Supplement: Table S3 — The summary of analyzed-sequence information with samples collected at the ophthalmology practice. [file mbo002162742st3.pdf]

**Table S3. The summary of analyzed sequences information with samples collected at ophthalmology practice.**

| **Sampling Site** | **Ophthalmology practice** | | |
|---|---|---|---|
| **Wearers Type** | **Non-lens wearers (n=22)** | **Lens wearers (n=16)** | |
| **Sample Type** | **Conjunctiva** | **Conjunctiva** | **Total** |
| No. of samples | 43 | 32 | 75 |
| **Total no. of Seqs** | **989,786** | **771,070** | **1,760,856** |
| Mean no. of Seqs (± Std dev) | 23,018 ± 12,195 | 24,096 ± 16,500 | 23,478 ± 14,202 |
| **Total no. of Seqs yielding OTUs** | **958,587** | **743,616** | **1,702,203 (3.3% loss)** |
| Mean no. of Seqs yielding OTUs (± Std dev) | 22,293 ± 11,919 | 23,238 ± 15,780 | 22,696 ± 13,708 |
| **No. of observed OTU types** | **3,057** | **2,988** | **4,573** |
