# Supplementary material for: Changes in the Eye Microbiota Associated with Contact Lens Wearing
Source: mBio. 2016 Mar 22;7(2):e00198-16. doi: 10.1128/mBio.00198-16 (PMC4817251; doi:10.1128/mBio.00198-16)
Supplement: Figure S5 — Bacterial diversity in the conjunctiva, skin, and contact lens samples by gender. Unweighted UniFrac distances were used to evaluate diversities between samples. (A and B) PCoA plots of bacterial communities in conjunctiva and skin under the eye of non-lens wearers (A) or lens wearers (B). The significant differences in PCoA plots were obtained using PERMANOVA. (C and D) Histogram of unique biomarker conjunctival bacteria in each gender of non-lens wearers (C) or lens wearers (D). The LDA effect size (>3.0-fold) was used to detect unique biomarkers. (E) PCoA plot of bacterial communities classified by lens wear and gender. (F) Box plots of intergroup distances in bacterial communities between conjunctiva and skin under the eye. Download [file mbo002162742sf5.pdf]

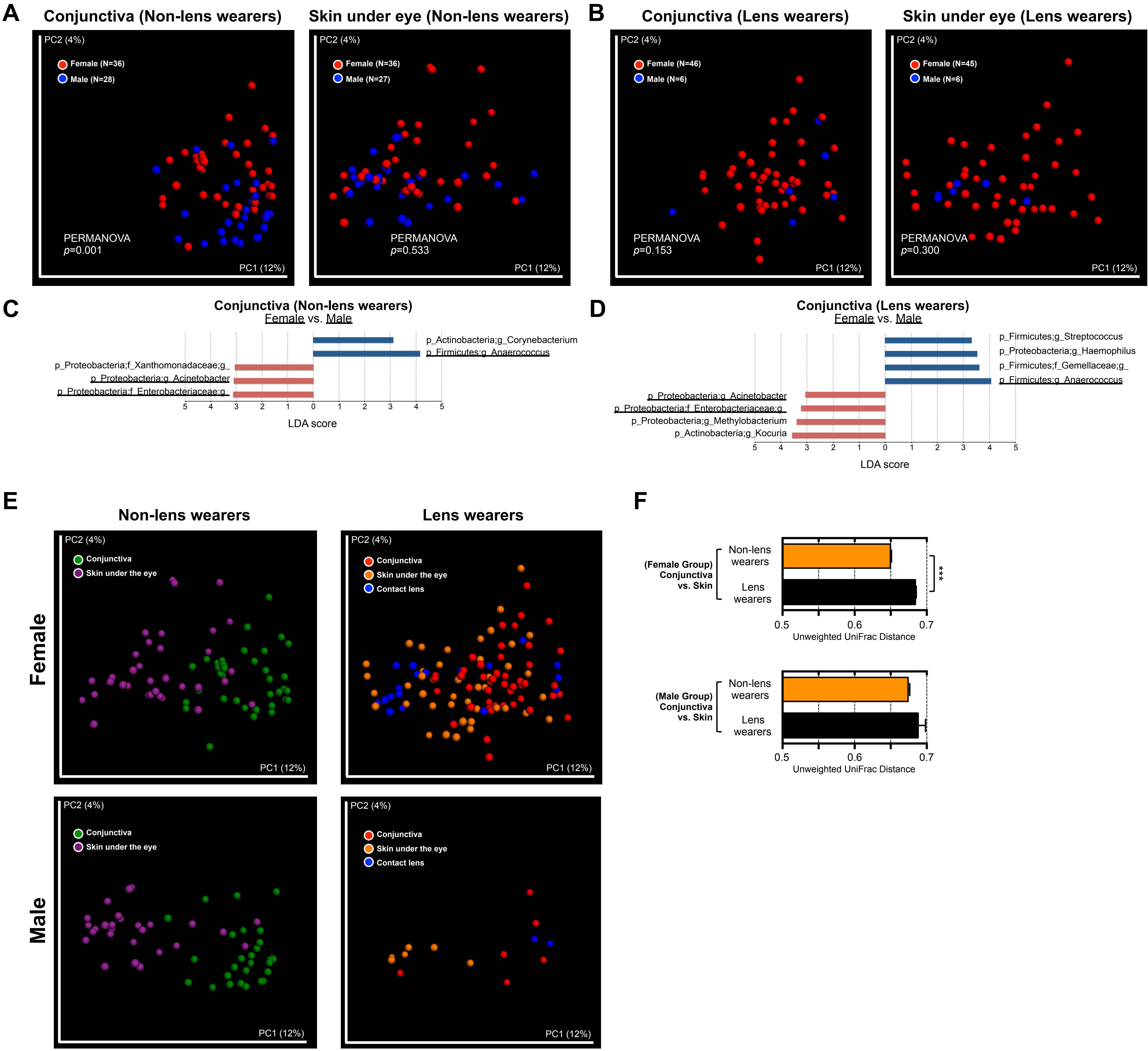
A
Conjunctiva (Non-lens wearers)
Skin under eye (Non-lens wearers)
B
Conjunctiva (Lens wearers)
Skin under eye (Lens wearers)
PC2 (4%)
PC1 (12%)
Female (N=36)
Male (N=28)
PERMANOVA p=0.001
Female (N=36)
Male (N=27)
PERMANOVA p=0.533
Female (N=46)
Male (N=6)
PERMANOVA p=0.153
Female (N=45)
Male (N=6)
PERMANOVA p=0.300
C
Conjunctiva (Non-lens wearers)
Female vs. Male
p_Actinobacteria;g_Corynebacterium
p_Firmicutes;g_Anaerococcus
p_Proteobacteria;f_Xanthomonadaceae;g_
p_Proteobacteria;g_Acinetobacter
p_Proteobacteria;f_Enterobacteriaceae;g_
LDA score
D
Conjunctiva (Lens wearers)
Female vs. Male
p_Firmicutes;g_Streptococcus
p_Proteobacteria;g_Haemophilus
p_Firmicutes;f_Gemellaceae;g_
p_Firmicutes;g_Anaerococcus
p_Proteobacteria;g_Acinetobacter
p_Proteobacteria;f_Enterobacteriaceae;g_
p_Proteobacteria;g_Methylobacterium
p_Actinobacteria;g_Kocuria
LDA score
E
Non-lens wearers
Lens wearers
Female
Male
Conjunctiva
Skin under the eye
Contact lens
F
(Female Group) Conjunctiva vs. Skin
(Male Group) Conjunctiva vs. Skin
Non-lens wearers
Lens wearers
***
Unweighted UniFrac Distance
